# Public health, conflict and human rights: toward a collaborative research agenda

**DOI:** 10.1186/1752-1505-1-11

**Published:** 2007-11-15

**Authors:** Oskar NT Thoms, James Ron

**Affiliations:** 1 Independent research consultant, Ottawa, Canada; 2 Norman Paterson School of International Affairs, Carleton University, Ottawa, Canada

## Abstract

Although epidemiology is increasingly contributing to policy debates on issues of conflict and human rights, its potential is still underutilized. As a result, this article calls for greater collaboration between public health researchers, conflict analysts and human rights monitors, with special emphasis on retrospective, population-based surveys. The article surveys relevant recent public health research, explains why collaboration is useful, and outlines possible future research scenarios, including those pertaining to the indirect and long-term consequences of conflict; human rights and security in conflict prone areas; and the link between human rights, conflict, and International Humanitarian Law.

## Introduction

In fall 2006, a controversial estimate of Iraqi war deaths published in the *Lancet *[1] made world headlines, spurring a renewed round of debate over the ethics and consequences of the US-led Iraq invasion. The survey found that some 650,000 Iraqis were likely to have died as a result of the insurgency and sectarian strife following the 2003 invasion. The political ramifications of this figure were undeniable, given US leaders' insistence that their invasion had been, in part, motivated by humanitarian considerations [2]. Yet the report also became a magnet for critics, with many questioning the study's baseline assumptions, sampling methods, and data reporting procedures [3-6].

Methodological criticisms aside, the *Lancet*-inspired media furor clearly heralded the growing impact of public health research on conflict and human rights analysis. In particular, it drew attention to the capacity of "conflict epidemiologists" to provide science-based estimates of the direct *and indirect *cost of war. Most importantly, perhaps, these epidemiologists are gradually demonstrating that most existing studies grievously under-estimate war's overall human cost by failing to capture its indirect and long-term impacts [7-11]. From a human rights perspective, moreover, the legal liability of the commanders and politicians responsible for this collateral damage remains uncharted territory.

Epidemiological studies can also generate important evidence for policy decisions, as witnessed in the case of the Democratic Republic of Congo (DRC), where surveys by the International Rescue Committee (IRC) have called attention to the country's ongoing humanitarian crisis by discovering vast numbers of indirect, war-related deaths [12-15]. Of 3.9 million excess deaths from 1998 to 2004, according to these surveys, only a small proportion have been directly related to political violence, with the remainder attributed to war-related ailments, such as disease. These findings have proved influential in policy circles, boosting the conflict's international profile while enhancing the resources available to peacekeepers and aid workers alike [comments by representatives from the IRC, Human Rights Watch, and Catholic Relief Services, at workshop on "Integrating Public Health Methods and Data into Conflict Analysis," Ottawa, March 9, 2007].

Public health research has also helped assess harmful policy impacts short of armed conflict. One case in point is the 1999 study by a Columbia University researcher on the link between sanctions and Iraqi child mortality, which revised previous estimates downwards while still confirming that childhood mortality had risen at alarming rates [16]. These and other findings, according to one UN insider, played a key role in curbing the international body's appetite for comprehensive embargoes [comment by Andrew Mack (former head of strategic planning for the UN Secretary General), at workshop on "Integrating Public Health Methods and Data into Conflict Analysis," Ottawa, March 9, 2007].

Finally, consider another 1999 study of mortality, this time in the Serbian province of Kosovo, which argued that 12,000 people likely died during the conflict between Serbia, NATO and the Kosovo Liberation Army [17]. By mapping trend data against key political and military events, the report demonstrated that Serbian military activities, rather than NATO air strikes, were correlated with spikes in mortality. This study broke new ground by linking survey research to international humanitarian law (IHL, or the "laws of war"), and its findings have found their way into deliberations at the International Criminal Tribunal for the former Yugoslavia [18]. More than any other study, perhaps, this analysis illustrates the common interests of scientifically rigorous public health researchers, policy-oriented conflict analysts, and human rights monitors, underlining the value-added by multi-disciplinary research.

Although not all public health research is of equal quality, this article argues that closer collaboration is likely to continue to benefit epidemiologists, conflict analysts, and human rights monitors. Until now, public health's specialized methods, logistical complexities, and high costs have hindered multi-disciplinary research, and many non-specialists only dimly perceive opportunities for collaborative efforts. Many public health specialists, moreover, have a hard time demonstrating their value-added to lay audiences. This article seeks to bridge this gap by outlining epidemiology's utility for policy-makers, conflict analysts, and human rights monitors. For maximum effect, it should be read by non-epidemiologists in conjunction with a more technically oriented epidemiological primer [19,20].

## Epidemiology's potential contribution

Epidemiology is the statistical study of the distribution of health events, outcomes and risk factors. This paper focuses on one particular research tool: retrospective population-based surveys. This emphasis is not intended to identify epidemiology exclusively with survey methods, but reflects surveys' particular utility for assessing conflict and human rights impacts. Although real-time and accurate surveillance data from health care facilities often provide the best measures of current conditions, such data are rarely available in crisis zones. Retrospective surveys, which ask people to recall health events during a specified time frame, are a good way of bridging this gap. Epidemiology has no monopoly on survey research, and human rights groups also use retrospective methods; most, however, do not rely on population-based techniques.

Since it is rarely possible to survey an entire population (as in a census), researchers typically question samples that are, in theory, representative of larger groups. When samples are well designed, their measured characteristics should be similar to those of the population from which they are drawn. A retrospective survey thus involves a standardized, structured questionnaire about past events, an accepted sampling method, and a statistical procedure for inferring about the general population from the sample's findings. (The appendix provides a more detailed discussion of survey methodology.)

Historically, the field of conflict epidemiology emerged from public health research in humanitarian emergencies [presentation by Bradley Woodruff, at workshop on "The Epidemiology of Complex Emergencies," Ottawa, March 8, 2007]. Beginning in the early 1970s, researchers increasingly realized that epidemiology could help devise needs-based policies and make humanitarian assistance more effective, and in the 1980s and 1990s, epidemiological findings were used to create general recommendations for improving the effectiveness of humanitarian assistance. As a result, practitioner manuals have increasingly included guidelines on using epidemiological methods in humanitarian assessments. In recent years, experts have developed minimum standards for humanitarian aid [21] while standardizing epidemiological methods for assessing key indicators [22].

As noted above, conflict epidemiology has also proved relevant to general foreign policy debates, particularly those pertaining to the creation, deployment and effectiveness of humanitarian, peacekeeping and peace-building interventions. The quantification of sickness and death is a concrete measurement of the quality of life (or lack thereof) in insecure zones, and is easily understood by most policymakers, even if the specific research methods are not.

Although epidemiology is only one of several quantitative methodologies which can and should be used in assessing conflict and human rights conditions, it has great potential due to its ability to offer detailed knowledge about what is happening to people in conflict, and the immediate causes of those events. It can establish numbers and rates of health events within populations, and, importantly, identify risk factors for such outcomes in specific times and places. In sufficiently large samples, moreover, these data can be broken down by age, gender, ethnicity, caste, or region.

Health data is often of "dual use," informing both evidence-based humanitarian programs [23], as well as general policy, media and advocacy purposes. Crucially, epidemiological findings can be particularly useful to human rights monitors, since specific health risk factors may also be violations of human rights law or the laws of war. With proper sampling methods, epidemiology can give monitors a more accurate sense of how widespread particular violations may be, and when trend data are available, researchers can correlate health outcomes with key political, legal, or military events.

## NGO conflict analysis and human rights monitoring

Despite these clear advantages, population-based methods are rarely used by influential conflict analysis and human rights NGOs. Although the Boston-based Physicians for Human Rights (PHR) does a superb job of combining population-based surveys and human rights questions (see below), it is a small player in comparison to major NGOs such as Human Rights Watch (HRW) or the International Crisis Group (ICG). In 2005, according to annual reports, PHR's total budget was about US $4 million, compared to $11.4 and $26 million for the ICG and HRW, respectively. In 2006, moreover, PHR's 46 staffers were outnumbered by the ICG's 110 and HRW's 233. As a result of these discrepancies in size, PHR's comparative media impact is small. A keyword search of NGO names in the Factiva database, for example, found that in 2006, PHR was mentioned by *The New York Times *only seven times, compared to 63 and 157 for the ICG and HRW, respectively. These latter two NGOs are leading voices in global policy debates, and their research and advocacy is often considered "state of the art." To illustrate the value-added of collaborative research, we critically survey a small and non-random sample of HRW's and ICG's work.

HRW's and ICG's fieldwork is done at comparatively low cost, often with a slimmer field presence than epidemiological surveys would require. HRW typically sends a handful of researchers from its offices to record testimonies, often without explicit government permission. ICG staffers tend to be based more frequently within their countries of interest, but their research is similarly unobtrusive. Both groups rely on lengthy, unstructured interviews, but ICG's researchers focus more heavily on broader political and governance structures, while HRW concentrates on human rights violations. HRW generates new information on human rights abuses, but ICG sees its value-added as one of analysis and prescription.

### Problems of data use

Although reports written by the ICG and HRW are compelling, accessible, and effective, they would be even more powerful were they to rely on methodological input from public health researchers and other data experts. Consider a 2005 ICG report on forced urban displacements in Zimbabwe, which cited UN estimates of 700,000 displaced, and 2.4 million indirectly affected individuals. Although the ICG reported that its own "extensive research ... unearthed no basis for disagreement" with the UN data, it provided little information on either organization's methods [24]. The UN report itself, disappointingly, is similarly vague [25]. Further inquiry into the UN's data collection, as well as more reflection on the ICG's own research methods, would have strengthened the Zimbabwe report considerably. Had the ICG wanted to go one step further, moreover, it could have investigated the UN data in greater detail, examining its methods to ascertain whether the numbers were reliable and valid. Or it might have collaborated with survey researchers to generate new data on the forced displacement, including information on the health conditions of its victims. Did childhood disease climb after displacement? What was the displacement's impact on livelihoods, gender-based violence, and other key variables? This kind of information could have added much to our knowledge of the Zimbabwean displacement's impacts.

Consider also the ICG's 2006 report on Sri Lanka, which provided no source at all for its claim of "at least 70,000" having died in the country's north east over the course of the conflict, or for its assertion that over 2,500 persons had been killed since hostilities re-ignited in January 2006 [26]. Proper attribution and reflection on data quality are vital, especially for a widely read organization such as the ICG.

Similar problems are encountered in HRW's reports. Consider the group's 1995 account of violations of the laws of war in Turkey's Kurdish southeast, written by one of this paper's co-authors [27]. Although no widely accepted figures existed at the time, the report made use of a reputable local NGO's claim of two million displaced persons. It made no independent evaluation of that group's research methods, however, and presented few details for others to assess. The report also offered little sense of the displacement's impact on villagers' lives. For example, what effects did forced migration have on their health? Did they display high levels of mental trauma? Were they more likely to suffer from disease or child mortality? Retrospective surveys would have given readers and the Turkish public a better sense of the counterinsurgency's civilian impact.

Ten years later, HRW revisited the issue with a report disputing official figures on the extent of villagers' return [28]. The study was a laudable effort to delve into the nitty-gritty of official data, highlighting HRW's growing interest in the mechanics of quantitative work. To dispute the government's figures, HRW researchers visited several returnee villages, comparing local accounts of the extent of return to those of the government. Actual return figures, HRW found, were far lower than those claimed by the government.

### Problems of data collection

Yet while the 2005 report on Turkey was persuasive, HRW's evaluation of government statistics would have been further strengthened by more attention to sampling detail. For example, the 2005 report gave no information on how HRW researchers selected their village sample, saying only that researchers "visited a small sample of villages and hamlets" in three southeastern provinces [28]. As a result, its findings' broad applicability is difficult to assess. To address this problem, HRW might have visited a random sample of Kurdish villages drawn from an existing list of depopulated communities, and if that effort proved too laborious, the group could have used other accepted sampling techniques to select provincial village clusters, weighted by provincial population size. These and other methods would have strengthened the credibility and precision of the group's findings.

Unlike the ICG, HRW regularly generates entirely new data based on witness and victim testimony. The group's careful, one-on-one interviews are regarded as state of the art by human rights monitors, reducing potential bias through repeated probes and cross-validation. Yet many HRW interviews are carried out under adverse conditions, pushing its researchers to rely on non-random convenience samples, while in other cases, HRW builds its arguments around individual and noteworthy incidents. Both techniques are problematic. Purposive samples are useful for exploratory research and hypothesis building, and worst-case documentation is important for moral, advocacy and legal reasons. Neither, however, is well-suited to establishing a condition's overall prevalence. In seeking to move from samples to broader generalizations, HRW could usefully draw on the advice of epidemiologists and other quantitative researchers.

Consider HRW's 2005 report on Nigerian police brutality, which presented powerful testimonies from 50 persons abused in police custody over the previous four years. The report left little doubt that something was badly amiss in Nigeria's criminal justice system. Yet the report argued that "torture and other cruel, inhuman and degrading treatment by the Nigerian Police Force ... [is] widespread and routine," while simultaneously acknowledging that its researchers had focused "on a limited number of locations and cases" [29]. Respondents were interviewed in three separate regions of the country, but the report gave few details on how HRW researchers had chosen to interview these, as opposed to other, victims.

To strengthen the report's reliability, HRW might have adapted a standard sampling procedure. For example, HRW researchers might have taken lists from local Nigerian Bar Associations to generate a representative sample of defense attorneys in different regions, and these might have supplied HRW with names of recent clients willing to be interviewed. Although this procedure would have introduced some bias – not all detainees would be willing to speak to HRW, while others might not have access to lawyers – it would still have been a far more systematic approach to assessing the extent of Nigerian police brutality.

In adopting population-based techniques, however, HRW would have had to interview Nigerians whose police experience had been satisfactory, requiring a re-allocation of resources away from worst-case scenarios. Yet HRW, like most human rights groups, resists spending time and money on interviews with people who had no problems to report. Surveys, by contrast, are often obliged to expend enormous energies documenting a problem's non-existence. In the 2004 IRC study of mortality in DRC, for example, surveyors working on the International Rescue Committee study visited 19,500 households throughout the country, finding 4000 deaths in a 16-months recall period [15]. Although this finding implied an extraordinarily high national mortality rate, it also forced researchers to document far more *absences *of death than actual deaths. It is not clear whether a human rights organization such as HRW will be willing to use scarce resources in this fashion, even if the payoff is greater precision and credibility.

A final detailed example will suffice to illustrate the usefulness of collaboration. In 2006, soon after the end of hostilities, HRW produced a preliminary report on violations of the laws of war during the Israel-Hezbollah conflict in Lebanon [30], as well as two subsequent and more detailed reports on violations by Hezbollah and Israel [31,32]. The laws of war limit the right of belligerents to cause civilian suffering and prohibit efforts to destroy objects "indispensable to the survival of the civilian population" [33]. Incidental loss of civilian life in warfare is expected, but belligerents are obliged to limit collateral damage as much as they can. Determining the extent of IHL violations on both sides was a methodologically and legally complex affair. Both sides had rained thousands of rockets and shells on the opposite of the border, and both claimed that they were firing at legitimate military targets.

Given the political sensitivities involved, it is not surprising that HRW's analysis of Israeli violations attracted the most critical attention. At some level, of course, Israel's entire military effort in the summer of 2006 could have been regarded as illegal, since it destroyed so much Lebanese infrastructure while emptying such large swathes of civilian territory. HRW's analysis of IHL violations typically requires far greater precision, however, including sophisticated arguments about the legality of individual air and artillery strikes.

To determine whether particular Lebanese civilian deaths were the result of IHL violations, HRW had to first establish whether particular Israeli attacks were unlawful. The international legal principle of *distinction *holds that belligerents must distinguish between civilians and combatants, while that of *proportionality *demands force to be proportional and necessary. A careful IHL study, therefore, required painstaking, post-hoc reconstructions of Hezbollah activities in the target areas through conversations with witnesses and other informants, combined with nuanced analyses of Israeli intentions, capabilities and actions.

To conduct its study, HRW assembled lists of Israeli attacks that resulted in Lebanese civilian casualties. It then read media reports and spoke to key informants, seeking to determine which events allegedly involved indiscriminate fire, and then targeted this subset for more detailed field research. For the larger and more detailed 2007 report [32], HRW investigated the circumstances surrounding 561 (500 civilians and 61 combatants) of a total 1,109 Lebanese killed by Israeli fire. Almost 60% of the civilians killed, according to the HRW study, died as a result of unlawful Israeli strikes. As a result, HRW concluded that Israeli forces had systematically violated IHL [personal email correspondence with Iain Levine (HRW), September 20, 2007].

There is little question that HRW's research on this count was laudable, assembling important data under difficult conditions. Yet a population-based approach might have added still greater precision, helping HRW discern with even greater confidence whether Israeli violations had been both routine and widespread during the 2006 summer war.

For example, HRW might have first worked with local Lebanese authorities, medical workers and others to generate a reasonably comprehensive list of all communities targeted by Israeli fire (rather than just those where civilians died). Next, HRW might have sought to determine which of those communities had experienced civilian casualties. From this subset, HRW researchers could have then selected a representative sample, using accepted sampling techniques, for detailed field investigation. This sequence might have helped HRW better estimate the proportion of Israeli attacks involving IHL violations. The data could have then been disaggregated by time and region, giving a better sense of Israeli violations across time and space. This information, in turn, would have helped determine with greater precision the nature of Israeli culpability. For example, a small number of criminal attacks would suggest localized problems of coordination and control, while larger and more consistent patterns would indicate higher-level intentionality.

Finally, the report could have been usefully supplemented with health surveys or surveillance data from hospitals and clinics. With the help of public health specialists, HRW could have gained a better sense of the overall civilian impacts of Israel's campaign, which destroyed much of southern Lebanon's transportation infrastructure, homes and businesses. What, for example, were the maternal, child, and general mortality trends in the six months following Israel's campaign? Governments and armed groups should be held accountable for these indirect damages, an issue we return to below.

## What do conflict epidemiologists do?

Conflict epidemiologists are particularly concerned with conditions during and after complex emergencies, defined as "relatively acute situations affecting large civilian populations, usually involving a combination of war or civil strife, food shortages and population displacement, resulting in significant excess mortality" [34]. Mortality is a key indicator of overall population health [35], but epidemiologists may also seek information about a range of other health indicators, including morbidity, malnutrition, sanitation, and access to health care. Importantly, mortality is methodologically easier to assess than other health indicators. As noted above, mortality studies are important for assessing the direct and indirect impact of conflict, but this section also discusses other epidemiological research efforts relevant to conflict and human rights analysis.

### The causes and conditions of displacement

In some epidemiological studies, such as the Iraq *Lancet *study discussed above, epidemiologists survey *national *populations. Most conflict epidemiology, however, focuses on more compact and survey-able groups such as refugees or displaced persons. As a result, there is a dearth of good information about health conditions outside of clearly delineated population centers. Retrospective questionnaires can help address this gap by asking refugees or displaced persons about conditions prior to, and during, flight.

In Darfur, for example, survey researchers questioned villagers about events before, during, and after displacement, learning much about human rights and health conditions in inaccessible regions [36]. The surveys revealed that most respondents fled from militia violence, and that violent causes of death, rather than disease or hunger, predominated in the "village and flight" period. Once respondents reached organized camp locales, however, medical causes of death predominated, suggesting that respondents were largely safe from direct military violence. Thus even when it proves impossible to survey Darfur's interior regions, researchers can use retrospective surveys in safe peripheral areas to gather vital, science-based information on events in inaccessible zones.

Consider also a 1999 PHR survey in Macedonia and Albania among ethnic Albanian refugees fleeing Kosovo during the conflict between Serbia, NATO and the Kosovo Liberation Army. This study sought information on the time frame and reasons for displacement, and on experiences of human rights abuses. PHR could not send surveyors into Kosovo at the time, but surveys of refugees provided strong evidence that Serb forces had engaged in a systematic expulsion campaign [37].

### The civilian impacts of munitions and military tactics

Munitions impact studies are another powerful application of retrospective surveys. In a 1995 Mozambique study, for example, researchers found rates of landmine-related death and injury far in excess of those suggested by prospective surveillance methods [38], while in a larger study of landmine impacts in Afghanistan, Bosnia, Cambodia, Mozambique, surveyors found that six percent of households suffered landmine victims, and that 25–87% suffered landmine-related impacts [39]. In this case, retrospective surveys thus shed important light on the utility of a global landmine ban.

Such studies may also have important spill-over effects. In Afghanistan, for example, a study of landmine and unexploded ordnance impacts helped researchers launch a key informant strategy for estimating civilian deaths over large areas [40]. Using various data sources, surveyors visited all 747 Afghani communities suspected of having endured a coalition air or ground attack, finding 600 that had actually experienced hostilities. Rather than using a household survey, however, researchers elected to question local key informants in each community, with counterintuitive results: 43% of communities reported no direct-violence victims, while 66% had no landmine or unexploded ordnance deaths. Civilian casualties, in other words, were tightly clustered in a smaller number of locales, a finding the authors interpreted by differentiating between the impacts of air and ground attacks. NATO air raids appeared to scatter Taliban forces, leading to fewer civilian casualties; NATO ground attacks against Taliban fighters who held their ground or regrouped, by contrast, led to more civilian deaths.

The policy implications of this study were wide ranging; not only did it find that 5,576 Afghanis had been killed and 5,194 injured from September 2001 to June 2002, but it also shed light on the *way *in which these individuals had died. Methodologically, the study broke new ground by combining comprehensive key informant interviews with statistical techniques. The study located informants in *all *violence-affected communities, seeking to determine patterns and causes of death and injury. Although key informants may be biased by political affiliation or the desire for aid, the method's broad geographic coverage has clear practical and methodological advantages.

### Conflict-related morbidity

Epidemiologists also seek to estimate the effect of conflict on disease by using retrospective mortality studies called "verbal autopsies." The International Rescue Committee's surveys in the Democratic Republic of Congo, for example, found that infectious disease was the country's biggest killer, far outstripping direct conflict deaths and injury. Cross-national analysis of summary disease data has also found that civil wars greatly increase the risk of infectious disease [7]. The most important immediate causes of deaths in complex emergencies are acute respiratory infections, diarrheal diseases, maternal and neonatal morbidity, tuberculosis, and vector-borne diseases such as malaria. Disease risk is increased by several conditions common in complex emergencies, including overcrowding and inadequate shelter; malnutrition; insufficient vaccination; poor water and sanitation conditions; exposure to "new" diseases, for which affected populations have not developed immunity; and lack of, or delay in, treatment [41]. In recent years, researchers have also become concerned with the effect of conflict on particular communicable diseases, such as HIV-AIDS, but the links in this case remain contested [personal telephone communication with Paul Spiegel (UNHCR), December 5, 2006].

### Conflict-related mental health

Another use of population-based surveys lies in assessing the impact of complex emergencies on mental health. Although this remains a comparatively neglected area of study, the existing evidence suggests, not surprisingly, that mental illnesses increase in emergency settings, and that multiple human rights violations may have cumulative and negative mental health impacts [41]. Like indirect conflict mortality, adverse mental health impacts are part of a conflict's overall human costs, and should be factored into broader impact assessments.

Mental health impacts can also have important political consequences. Consider, for example, one study of links between traumatic experiences during the 1994 Rwandan genocide and attitudes towards post-conflict justice. Nearly a quarter of respondents displayed PTSD symptoms, and they were less likely to have positive attitudes toward the Rwandan national trials and interdependence with other ethnic groups. Furthermore, persons who experienced multiple traumatic events were more likely to have positive attitudes toward the International Criminal Tribunal for Rwanda but less likely to support national and local justice and reconciliation processes [42]. Consider also a study of mental health and attitudes among Kosovar Albanians following the 1998–99 war, which revealed an association between traumatic war time events, decreased mental health, impaired social functioning, and strong respondent emotions of hatred and revenge toward Serbs [43].

### The extent and scope of human rights abuses

As noted above, PHR has pioneered efforts to use population-based surveys in assessing the extent of human rights violations. In a number of cases, PHR's efforts have yielded important results. Research on sexual violence, for example, is inherently difficult [44]; PHR's 2002 report on the experiences of displaced persons in Sierra Leone, however, successfully produced a wealth of important data with the help of the local UN mission, trained local staff, and carefully designed surveys [45,46]. Seventeen percent of respondents in displaced person camps reported at least one lifetime sexual assault, while nine percent reported an assault during the war. And while this number appeared low given media reports of widespread sexual violence during Sierra Leone's civil war, PHR's survey established that the main rebel group, the Revolutionary United Front (RUF), was systematically committing sexual abuse. According to the study, 53% of the women reporting direct, face-to-face contact with RUF fighters also reported that they had been sexually assaulted, compared to less than six percent for those exposed to other combatant groups. As a result of these and other findings, the 2002 PHR report played a key role in Sierra Leone's transitional justice debates, pushing gender violence to the top of the agenda [47]. Another successful PHR study is its 2000 survey of displaced Chechens, which documented widespread abuse by Russian forces. In nearly all cases, PHR found, displacement was attributed to Russian actions, rather than those of Chechen insurgents [48]. PHR has conducted similarly innovative surveys on events in Kosovo (see above), Afghanistan, and Iraq.

Another example of inter-disciplinary research comes from an innovative Johns Hopkins team that has found a correlation between human rights violations and specific adverse health outcomes [49]. At the initiative of local "back-pack" medics working in Burma's eastern border area, researchers inserted a series of human rights questions into a 2004 health survey. Of 1,834 surveyed households, 33% reported being subjected to forced labor, nine percent had been internally displaced, and 25% had food or other essential items stolen or destroyed by Burmese military forces. With the help of these findings, the team was able to compare the health of displaced and non-displaced families, finding that the former were 2.8 times more likely to have experienced a child's death, 3.2 times more likely to have a malnourished child, and 3.9 times more likely to have suffered a landmine injury. Those experiencing human rights violations, moreover, were also more likely to experience child mortality and landmine injury. By correlating specific health problems to specific abuses, the Johns Hopkins researchers successfully provided evidence useful to human rights monitors, humanitarian workers, and conflict analysts alike.

### Post-conflict conditions

Population-based surveys have also provided information about conditions in post-conflict settings. Although peace should theoretically be associated with greater physical and mental well-being, this is not always true. For example, PHR studied health conditions in Chiapas, Mexico, years after insurgents ended their armed rebellion [50], and their survey of 2,997 households in 46 communities discovered that health conditions had in fact deteriorated alarmingly, with some communities being denied healthcare for political reasons. Thus, while Chiapas' shooting war had ended, health conditions were in fact getting worse, not better. Unfortunately, researchers may find similar post-conflict deterioration elsewhere.

At the policy level, these and other findings strongly suggest that the UN and other agencies should commission immediate post-conflict surveys to establish baseline data on existing human rights and health conditions. Over time, follow-up studies could then track improvements, or lack thereof, for specific population segments. This combination of baseline and follow up research could then give scientists, human rights activists, and policy makers reliable information on the real impacts of post-conflict arrangements on public health and well-being.

## The limits of population-based surveys

Like any research method, retrospective surveys suffer from limitations, and they are neither useful nor appropriate for all times and places. For starters, population-based surveys are logistically complex and costly, requiring local teams of trained researchers, coordination and supervision. Epidemiology is a highly technical affair, requiring training and experience in sampling, questionnaire design, interviewing, and statistical analysis. Given these and other complexities, it is not surprising that experts often criticize field NGOs' surveys [51]. Keeping up with the methodological state of the art is difficult, and experts continue to refine accepted techniques. Experts, moreover, constantly debate the most appropriate methods for different settings [36,52,53]. Complex emergencies vary dramatically, and a one-size-fits-all research method is not appropriate [35].

Population-based surveys are often difficult to implement in insecure areas, since both survey teams and respondents are vulnerable and hard to monitor. Governments or armed groups frequently deny access, making studies difficult where they are needed the most. Importantly, surveys in politically tense environments can raise thorny ethical dilemmas by placing both informants and researchers at risk of reprisals or re-traumatization [54-58]. If epidemiology is increasingly used for human rights analysis and to provide grounds for external intervention, moreover, governments may begin to block general public health research among needy populations, to the detriment of humanitarian assistance programs. [personal email communication with Francesco Checchi, November 6, 2006]. Yet the failure to use powerful research methodologies for advocacy on behalf of vulnerable populations may itself be unethical [54].

The survey process is also vulnerable to political manipulation from all sides. For instance, asking respondents about who is responsible for individual deaths is problematic, as respondents may give false information for a wide variety of personal and political reasons. Respondents may also not be willing to tell interviewers that members of their households were combatants. Another ethical issue arises if everyone involved in a survey, including researchers employed by humanitarian agencies, has an interest in inflated numbers. For this reason, many experts believe that scientific data collection and political advocacy should be kept separate to maintain the science's legitimacy and credibility [participant comments at workshop on "Integrating Public Health Methods and Data into Conflict Analysis," Ottawa, March 9, 2007]. These issues should not preclude collaboration between epidemiologists and conflict analysts/human rights monitors, but they do need to be addressed in the research process.

A final drawback of epidemiological research is that the relevance of its findings can be difficult to convey to policy-makers and the general public. As the polemic inspired by the Iraq *Lancet *study suggests, the media's agenda may focus too heavily on perceived methodological problems, despite poor understanding of the technicalities involved, and of these problems' implications for the results' validity. Policy-makers opposed to a given study's findings will dismiss them as imprecise, while advocates may fail to acknowledge that their numbers come with biases and substantial margins of error.

## Why collaborate?

While epidemiology is a powerful and under-exploited tool, the quantification of suffering is rarely sufficient, on its own, to ensure action. The political, economic, and logistical barriers to effective external intervention are well known, while new research has emerged suggesting that there are also substantial psychological barriers to promoting better public awareness of, and concern for, mass atrocities [59]. Full exploitation of epidemiology's potential will thus require close collaboration between public health analysts, conflict researchers, and human rights monitors.

There is little doubt that the research and writing styles of large NGOs, such as HRW and the ICG, offer important advantages, including unobtrusive research in insecure areas, and broadly accessible, easy-to-read reports. More importantly, perhaps, their detailed, confidential interviews with officials and other key informants can help establish causality in ways that statisticians find hard to emulate. Although epidemiology can demonstrate correlations, precise causal links are often more easily revealed through qualitative methods, such as "process tracing" of political decisions, chains of command, and actors' intentions, which "is fundamentally different from statistical analysis because it focuses on sequential processes within a particular [...] case, not on correlations across cases" [60].

While all organizations using data should understand and communicate its limitations, we are particularly concerned with the work of organizations generating new data, such as HRW. Like most human rights groups, HRW's ethos is grounded in international law, and most of its employees are not trained in epidemiology or other quantitative methods. With limited staff and a host of pressing demands, HRW finds it hard to prioritize discussions of careful sampling and data collection. Still, the group is constantly re-examining its research methods, and innovative collaborative efforts are already underway, including the group's report on abuses in Kosovo, in which data from 577 witnesses was coded and analyzed [61], and its report on Bangladeshi police forces, which charted the distribution of killings per population across police divisions [62].

Qualitative research groups such as HRW are not likely to transform themselves into survey outfits in the near future. Still, HRW and other qualitative research groups can and should become more conscious of their methodological limitations. Conflict epidemiologists, among others, can help generate more scientifically defensible evidence, and can also help clarify what the evidence shows, and what it does not. Although neither HRW nor the ICG have voiced interest in creating an in-house epidemiological capacity, both have expressed an interest in public health collaboration, including joint questionnaire design and better use of existing epidemiological results. At the same time, we discern growing interest among public health researchers in broader dissemination of their methods and data, and in working with others on the underlying causes of conflict and human rights abuse [34,63,64]. To be effective, these different research communities should become more literate in each other's lexicons, and engage in more frequent and respectful collaboration. The time for new research partnerships has arrived.

In this paper, we have provided a number of examples of public health research with proven relevance to conflict and human rights analysis. We conclude with a final collaborative scenario: the application of IHL analysis to the long-term human costs of war. At present, IHL offers little commentary on the legality of destroying the public infrastructure necessary for long-term health and human rights, preferring to concentrate on war's shorter-term and more immediate effects [65]. International lawyers find IHL's proportionality principle particularly hard to apply over time due to the intervention of complicating factors that make it hard to link cause and effect. Twelve months after a war's end, how much of a country's increased infant mortality could realistically – and legally – be attributed to wartime actions by combatants, as opposed to those of myriad other actors and events?

Given these complexities, human rights groups have hitherto preferred to focus on shorter time frames, where causality and legal responsibility are easier to establish. Over time, however, the accumulation of good quality epidemiological data can help broaden and extend the IHL analysis to longer post-conflict periods. The availability of relevant information is crucial, since IHL violations are judged on *expected *losses weighed against *anticipated *military advantages. As one analysis notes, "it is unacceptable for the expected military advantage to be based on a longer timeframe while limiting the expected quantification of civilian damage only to the immediate effects of the attack itself" [66]. By repeatedly documenting the short, medium and long-term impact of specific military tactics, epidemiological research can force military planners to increase the horizon of what they can reasonably predict. This argument is already being used in the ongoing debate over cluster munitions, where some believe IHL requires commanders to consider the explosives' long-term threat to civilians [66].

## Conclusion

Epidemiology is able to provide evidence of human suffering of great value to conflict analysts and human rights monitors. More often than not, information on the civilian impacts of conflict is based on informed guesses by NGOs and multilateral organizations, rather than rigorously assembled scientific data. This paper has identified problems of data use and collection by two major advocacy NGOs, arguing that these short-comings are particularly problematic when establishing the overall prevalence of a particular human rights abuse or conflict pattern. These data gaps, we argue, can be addressed in part through greater collaboration with public health researchers.

Epidemiology can help quantify the differential direct and indirect impact of conflict on particular populations, while trend data can track impacts over time, enabling researchers to map health outcomes against major offensives; peacekeeping operations; humanitarian assistance flows; and peace agreements. This information can shed light on the efficacy of international engagement in conflict zones, while providing human rights investigators with a way of assessing the extent and impact of violations across populations.

Research collaboration between public health specialists, conflict analysts and human rights monitors faces practical and ethical difficulties. These should be acknowledged and addressed, but they should not preclude the kind of collaborative research that could benefit needy and distressed populations.

## Appendix: How are population-based surveys done?

Epidemiological surveys collect quantitative health indicators from populations at a specific time, using standardized, structured questionnaires. In retrospective surveys, surveyors ask respondents to recall health events that occurred during a specified time frame known as the *recall period*. Although surveys can be exhaustive by including every person in the population (such as a census), they are usually based on representative samples. When samples are well designed, their measured characteristics should be similar to those of the population from which they are drawn. Survey design has to contend with bias (non-sampling error) and imprecision (sampling error).

Sampling is the selection of a specified number of persons or households from a population. Epidemiologists usually employ *probability sampling*, which ensures that every selected person or household has the same known chance of inclusion. Sample size should be large enough to provide reliable estimates, but not so large so as to waste limited time and resources. Larger samples are required for greater statistical *precision *or to investigate a condition with low prevalence within the population, such as maternal mortality. There are three general methods of probability sampling.

*Simple random sampling *requires a complete list of all the units to be sampled, such as households, and a certain number are then randomly selected from this *sampling frame*. Although this method is often the most representative, it is rarely feasible in conflict settings because of the paucity of complete lists. But even if good listings are available, simple random sampling is generally more expensive as it requires broad coverage of wide areas, and is thus logistically complex. For these reasons, simple random sampling is often only used when studying registered populations that are concentrated in small areas, such as well-organized refugee camps.

*Systematic sampling*, by contrast, randomly selects only the starting unit; all other units are selected by adding a certain number (known as the *sampling step*), which depends on the desired sample size. While this method does not require a comprehensive list to start, it does need a well-ordered population and a good estimate of population size, so that the sampling step can be calculated and applied. Again, systematic sampling is often possible in refugee camps or other well-delineated populations.

A third method, *multi-stage cluster sampling*, begins by listing clusters of sampling units, such as administrative divisions or villages, and then randomly selects a certain number of these. Cluster selection must be proportional to relative population size, so that areas with greater populations are allocated more clusters. Clusters can be selected at more than one stage of sampling. At the final stage, variants of the sampling methods outlined above can be used to select an equal number of households from each cluster. In many cases, the first household is selected randomly, while the rest are selected by proximity to the first. This method is a good way of creating representative samples even when there are no adequate listings of the entire population, or when households are not distributed in an ordered pattern. To do a good multi-stage cluster sample, one must simply be familiar with basic geographic divisions and their relative population size. Cluster sampling may also limit logistical and security concerns by reducing the movement of survey teams to a few random points. This also makes cluster sampling cheaper than random sampling. For these reasons, cluster sampling is often the sampling method of choice in complex emergencies.

Multi-stage cluster sampling has important drawbacks, however. Cluster sampling cannot be used to analyze quantitative differences between geographic divisions unless the population is first stratified by relevant criteria, with separate cluster samples drawn from each stratum. This increases the overall number of clusters needed. Moreover, statistical precision is lower in cluster samples, since households within clusters are more likely to resemble each other than if they were selected randomly from the entire population. This leads to a loss in sampling variability known as the *design effect*. This is particularly problematic when measuring highly clustered phenomena such as the effects of violent conflict. To compensate, researchers must increase the sample's overall size. Since it is statistically preferable to increase the number of clusters rather than the number of households within clusters, this compensatory adjustment often boosts the survey's cost and duration.

Samples always come with *biases*, which should be minimized and acknowledged. To prevent avoidable biases, researchers must try to ensure that the data they collect closely reflects the respondents' situation. This requires that the data collection effort be standardized and tightly monitored for quality. Questionnaires should be simple and clear, and fewer questions generally provide better measurements. Survey interviewers should be identically trained so that they do not influence responses.

## Competing interests

The author(s) declare that they have no competing interests.

## Authors' contributions

ONTT carried out the literature review, participated in the design of the study, drafted the initial manuscript, and is principal author. JR conceived of the study, obtained the funding, coordinated the workshop, designed the study and manuscript structure, contributed some sections, and edited the manuscript. Both authors participated in revisions and read and approved the final text.
